# Consensus on priorities in maternal education: results of Delphi and nominal group technique approaches

**DOI:** 10.1186/s12884-019-2382-8

**Published:** 2019-07-24

**Authors:** Carmen Paz-Pascual, Isabel Artieta-Pinedo, Gonzalo Grandes, ema.Q Group, Sonia Alvarez, Pilar Amorrortu, Maria Berruezo, Mónica Blas, Inés Cabeza, Joaquina Dueñas-Gil, Itziar Estalella, Edurne Estévez, Ana Cristina Fernández, Marie Pierre Gagnon, Gloria Gutierrez-de-Terán-Moreno, Kata Legarra, Mario López-Mateo, Gorane Lozano, David Moreno-López, Maria Jesús Mulas, Alba Padró, Covadonga Pérez, Angela Rodríguez, Mercedes Saenz-de-Santamaría, Jesús Sánchez, Maria José Trincado

**Affiliations:** 1 Atención Primaria en Salud, Prevención y Enfermedades Crónicas, IIS Biocruces Bizkaia, Plaza de Cruces 12. 48903, Barakaldo, Bizkaia Spain; 2 Midwifery Training Unit of Basque Country, Bilbao, Spain; 3 Faculty of Medicine and Nursing, University of the Basque Country UPV/EHU, Leioa, Spain; 4 Biscay Primary Care Research Unit (UIAPB), Osakidetza, Bilbao Spain; 5 OSI Barakaldo-Sestao, Osakidetza, Barakaldo Spain

**Keywords:** Prenatal education, Delphi technique, Health priorities

## Abstract

**Background:**

Maternal education is wide-ranging and covers many areas from pregnancy to the immediate postpartum period and childrearing. However, for it to be effective, more resources need to be assigned to key topics. The goal of this study was to identify and prioritize the most important issues in maternal education, so that specific objectives could subsequently be set and learning outcomes evaluated.

**Methods:**

We drew up a comprehensive list of topics addressed in existing maternal education programs, based on a systematic review of information obtained from the Internet and the experience of the research team. The topics were presented to a multidisciplinary panel whose members were asked to rate them from 1 to 9, and consensus of opinion was reached using a two-round Delphi survey, with consensus defined beforehand as 80% agreement among panelists in awarding a score of 7, 8 or 9. The most highly-rated topics were then discussed and again prioritized by a multidisciplinary team of healthcare and non-healthcare experts, using a nominal group technique.

**Results:**

Initially, 650 topics were identified and grouped into 80 categories which were then prioritized by 54 healthcare and non-healthcare experts using a Delphi survey with a study participation rate of around 20%. 63 topics were considered very important, so criteria were restricted and only the 24 highest-scoring selected (95% of agreement on scores ≥7 or 80% of agreement on scores ≥8). Using the nominal group technique, a group of 12 experts identified the following priorities: initiation and establishment of breastfeeding, development of a birth plan, identification of problems and self-care postpartum, nutrition and a healthy lifestyle, options for pain management in labor and birth and characteristics of a normal newborn/looking after a newborn baby.

**Conclusion:**

This study, with a Delphi study and the Consensus among Experts: the nominal group technique, has succeeded in identifying priority topics in maternal education. We need to assess women’s needs in relation to these topics, design an intervention to respond to these needs and evaluate its effectiveness.

## Background

Maternal education is a complex process aimed at enabling women to obtain knowledge, skills and emotional support so that they can take care of themselves and their child during pregnancy, the postpartum period and childrearing [1]. Designing effective support for mothers means involving the people it targets from the outset [2, 3], given that if the mother feels that her needs are met, her feelings of control and satisfaction will be greater [4], any health problems are more likely to be detected early and she will make fewer unnecessary visits to healthcare centers [2, 5]. Nowadays, women feel responsible for their own healthcare and demand a type of care from health systems that focuses on their needs [5–7] and also respects their values, expectations and circumstances during pregnancy, childbirth, the postpartum period, breastfeeding and childrearing [7–9]. Among these demands, there is a desire for health professionals to provide continuous support and information that is accurate, up-to-date, tailored to their needs and available in real time [9–11].

Various different studies have found that the information requirements expressed by women during pregnancy and the postpartum period include information concerning warning signs [10], what can they do to improve their health and that of their child [9], care of the infant [11], physical and emotional changes in the postpartum period [12] and resources available in the community [13]. Consequently, maternal education sessions tend to address all the aforementioned topics, as well as other issues.

However, attempting to address these matters with limited time and space makes it difficult to deal with particularly relevant topics in depth. In addition, the importance of each need varies over time and may differ between cultures [13–15]. Perhaps as a result, women often feel that their information needs during pregnancy are not fully covered by what they receive from the healthcare system [13, 16–20]. To increase the effectiveness of any intervention, the first step is the identification of the priority areas to which more resources should be devoted [15, 21, 22] and then the design of specific action strategies and evaluation of the results [23–27]. Therefore, in the first thing, it is necessary to answer the question: to which topics, in maternal education, do we need to dedicate most of the resources?.

The objective of this study was to identify and prioritize the topics that are the most important from the point of view of women and the health professionals who care for them, with the final goal of ensuring that any new maternal education programme provides an effective response to women’s needs, thereby increasing their ability to make their own decisions.

## Methods

In order to identify the topics for special attention during pregnancy, labor, birth, the postpartum period and childrearing, we carried out a mixed methods study in three phases: 1.- a comprehensive description of content related to pregnancy, labor, birth, postpartum, breastfeeding and childrearing addressed in existing maternal education programs and parenting websites; 2- prioritisation of topics using the Delphi method; and 3.- selection of the five highest priority topics, by seeking consensus among experts, using the nominal group technique. The combined use of these two approaches should allow us to reduce any potential bias arising from each independently [28, 29].

The protocol for the study was approved by the Clinical Research Ethics Committee of the Basque Country (PI2012072).

### Setting

This study was carried out in the Basque Country, which has a population of 2.176.016. Osakidetza - Basque Health Service provides universal free health care, which is fully financed by public funds. In 2017 there were 17,150 births and the average age of child-bearing women was 33.4. Midwives and obstetrician/gynecologist monitor pregnancies and offer maternal education to all women in primary healthcare centers, and 88.12% of deliveries occur in public hospitals, where the cesarean rate is 13%, while cesareans make up 26% of deliveries in private hospitals [30]. All primary care midwives are aware of the protocols followed in these hospitals, which prevents discrepancies between the maternal education provided and the subsequent situation in the hospitals. The services provided in these hospitals (e.g. epidural anesthesia) are offered to all women; midwives attend normal births, while obstetrician/gynecologist intervene in instrumental deliveries, multiple births, and breech presentations. Postnatal care within hospitals is carried out by nurses, not midwives, in most Spanish hospitals.

#### Description of the potential content on pregnancy, labor, birth, postpartum and childrearing

Potential topics to be addressed in maternal education were obtained, on the one hand, from the professionals who deliver current maternal education, midwives, who are represented in the research team by 10 midwives who offer maternal education to women in primary healthcare Osakidetza - Basque Health Service. And on the other, from websites accessed by women seeking information during pregnancy and early motherhood [31–33].

We carried out a systematic review of the most popular Internet search engines, Google, Yahoo and Bing [34], using the keywords: *embarazo*, pregnancy, *parto,* childbirth, *puerperio,* postpartum, *lactancia,* breastfeeding, bottle feeding, “*bebé* O *recién nacido*”, “baby OR newborn”, *educación maternal and* “antenatal education OR maternal education”. We then selected the 25 first websites listed by each search engine, identifying a total of 975 links. We also examined the resulting websites using the LIDA questionnaire, [35] version 1.2, which analyzes website reliability in terms of whether websites are up-to-date, whether there are conflicts of interest and using references about content production. We selected those with “good” or “excellent” reliability [36]. Each website was reviewed independently by two researchers, who created a systematic list with all the topics addressed. On the basis of this comprehensive list, we produced a questionnaire that included these topics, leaving a blank space at the end so that participants would be able to add any other topics they considered important. To assess whether it was written in language that would be easily understood by members of the public not involved in healthcare, the questionnaire was reviewed by three sociologists and three journalists (experts in communication).

#### Prioritisation of topics using the Delphi method

We used the Delphi method to obtain the most reliable consensus of opinion of a group of experts, because it enabled us to consult the opinion of a large number of anonymous people and gave them the opportunity to confirm their initial response or change their opinion. In this study, we sent out an electronic questionnaire and used two rounds [37–39].

##### Selection of participants

A convenience sample of experts with experience in women’s health and childcare was recruited from various parts of Basque Country. This project is part of a wider project that started evaluating the utility of maternal education and analyzed women’s needs during pregnancy, birth and after birth. [40] The analysis of the necessities was made focusing on pregnant and puerperal women groups [9]. This work moves on this line, focusing on the need of information, it aims to identify the topics that need more resources. To represent the opinion of mothers, we contacted non-health experts who, in addition to their individual experience as mothers, could offer a broader perspective because of their work with groups of women close to motherhood and they were aware of their needs globally, such as 42 representatives of women’s associations that had more than 300 members, were from ethnic minorities, or were directly related to pregnancy, birth and/or the postpartum period [41]. We also contacted the heads of 46 nurseries and 151 pre-schools (for children under 2 years of age). Then we selected 117 health professionals who provide care for women from the preconception stage until the end of the puerperium. These health professionals were selected by the research team, which itself is made up of a large number of healthcare professionals (*n* = 20) from different levels of care. The members of the team identified other professionals who in their opinion had relevant expertise, based on their length of practical experience and track record in innovative clinical practice, research and/or teaching and who were interested in this study. The 117 healthcare professionals proposed consisted of 70 midwives, 14 primary care-based pediatric nurses, 4 hospital-based postnatal nurses, 13 obstetrician/gynecologist and 16 pediatricians.

##### First round of the Delphi study

We sent an email with a questionnaire to candidate participants in September 2016. We attached a letter introducing the study, an invitation to participate and instructions for completing the questionnaire; it was essential to complete the first page with the informed consent form to be able to access the list of topics. The participants accessed the on line questionnaire with a code, so their completed questionnaire was anonymized. Each of the topics indicated were followed by a scale from 1 to 9, the topics rated 1, 2 or 3 being considered “unimportant”; 4, 5 or 6 “unsure” and 7, 8 or 9 “important” [38, 39].

Two weeks after the first email, we sent a reminder and a second invitation to participate to those who had not responded within this period. Only questionnaires received up to 3 weeks after the first mailing were included in the subsequent analysis. All the completed questionnaires received were initially analyzed in Microsoft Excel and the median ratings for each item were calculated.

##### Second round of the Delphi study

Individuals who responded to the first round were sent a second questionnaire with the same items and two new columns, one showing how they themselves had rated each item and the other showing the panel’s median rating. As had been explained in the letter introducing the study, they were invited to confirm their initial ratings or modify them, in the light of the opinions of the rest of the panel. Once again, a reminder was sent to people who did not respond in the first 2 weeks, and only completed questionnaires received within 3 weeks were included in the analysis.

##### Quantitative analysis

We calculated the percentages of participants that rated each item as unimportant (scores ≤3) or important (scores ≥7). We considered there to be a consensus when 80% of participants agreed on a topic being unimportant or important. All items agreed to be unimportant were removed, while in the case of items considered important, as most participants could be expected to rate most items highly, we also analyzed the percentages of individuals that rated items with scores of 7, 8 or 9 separately. We also assessed the potential differences between healthcare and non-healthcare experts by calculating Spearman’s correlation coefficient. All analyses were conducted using SAS statistical software (SAS PROC MIXED version 9.1, 2003; SAS Institute Inc., Cary, North Carolina) and *p* ≤ 0.05 was considered significant.

Free-text responses were registered and grouped into topics in order to identify any other issues considered important from the perspective of all the participants.

#### Seeking consensus among experts: the nominal group technique

Given that the topics listed are based on those usually addressed in maternal education sessions, it was expected that a large number of topics would be considered “important” by the expert panel. Moreover, the use of the nominal group technique in a study of this type makes it possible to analyze the results of the Delphi study in more detail, as it encourages debate, interaction and the generation of a wider range of ideas in situations when standard responses might not always be completely satisfactory [29, 42] The goal of the expert workshop was to select the five highest priority topics that need to be addressed by maternal education. The participants’ discussions were audio-recorded anonymously, with each participant assigned a code for the subsequent transcription. The recordings are stored in the Primary Care Research Unit of Basque Country and will be destroyed 5 years after the end of the study.

The workshop was presided over by two members of the research team. One of them acted as the moderator, introducing people, explaining the method and establishing whose turn it was to speak. The other was responsible for providing logistical support, the audio recording of the session, documenting the topics initially mentioned by each participant, and subsequently the ratings participants gave to the subset of the topics selected.

##### Participants

A convenience sample was selected among the Delphi study participants, who were offered the opportunity to discuss the issues raised in more detail. The group of experts were proposed by the research team, who debated the profile of the participants necessary to ensure that their areas of expertise covered all potentially relevant topics related to pregnancy, labor, birth, postpartum, breastfeeding and childrearing periods, to satisfy the following criteria:

- Eight healthcare professionals (2/3 of participants), who had at least 10 years of clinical experience and carried out their work in Basque Country, except in the case of home births:
  - Three primary care midwives, directly involved in the care of women during pregnancy, the postpartum period and childrearing, and in maternal education;
  - Two specialized-care midwives working in a hospital setting, involved with care of women in the delivery suite and after the birth until hospital discharge;
  - One midwife with experience in home births;
  - One hospital-based postpartum nurse, an expert in breastfeeding; and.
  - One primary care pediatrician;
- Four experienced non-health professionals (1/3 of participants), who offered a point of view that is closer to that of a recipient of maternal education, with an additional broader focus from their professional perspective and their own experience as mothers:
  - One journalist specializing in maternity issues who writes for a national publication;
  - Two individuals specializing in Information Technology (IT) applied to the promotion of maternal breastfeeding who had developed an online tool (www.lactapp.es);
  - One representative of a nationwide association, called “*El parto es nuestro*” (meaning “Birth is ours” in Spanish), which seeks to improve the conditions of care for women and children during pregnancy, labor, birth and the postpartum period in Spain (www.elpartoesnuestro.es).

The members of the panel of experts were invited by email, in which we explained the methodology of the workshop and the timetable. Once these individuals had agreed to participate, responding to the email sent, the research team sent them, again by email, the list of topics selected in the Delphi study and their qualifications, and also, once again, the complete Delphi questionnaire from the Delphi study listing all the topics.

##### Process

The *“Consensus workshop on the priority topics for maternal education based on women’s needs”* consisted of four 75-min sessions held on the same day in March 2018 in Bilbao. Before starting, participants were asked to consent to the audio-recording of the sessions and sign a confidentiality agreement. The nominal group technique was used in all the sessions, in which each participant proposed three topics which in their opinion were the most important and was given a maximum of 4 min to present, debate, and defend their position. Furthermore, although we started with a table listing the topics considered the most important in the preceding Delphi study, for their presentations, workshop participants could add any of the other topics from among the 56 that had been given scores ≤7 in the Delphi survey if they considered them important.

In the first session, participants discussed topics related to lifestyle and maternity in general, pregnancy and childbirth, selecting five priority topics. In the second session, they discussed topics related to the postpartum period, breastfeeding, childrearing, the newborn and young infant, again selecting five priority topics. In the third session, a consensus was sought on the five most important topics among the ten prioritized in the first two sessions. The five topics prioritized in each session were selected by consensus, or by vote if there was no clear majority preference concerning the most important. Each participant selected the five topics they considered a priority and ranked them by assigning 5 points to the most important topic and 1 point to the least important. Subsequently, the total sum of points obtained for each item was calculated, and the topics with the highest total scores were selected.

## Results

### Description of the potential content on pregnancy, labor, birth, postpartum and childrearing

The process used to obtain the complete list has been described in a previous paper [36]; in brief, a comprehensive list of 650 topics was obtained mainly from websites (350 topics) and was completed by adding other topics that were considered complementary or important by members of the research team: midwives, midwifery lecturers, pediatricians, nurses, and nursing lecturers (300 topics). Two members of the team first independently categorized items on the list and then reached a consensus on the categories, reducing the list to 83 topics. After further review, some topics were grouped so the final questionnaire contained 80 topics that could be addressed in maternal education. There were 10 topics concerning healthy lifestyle (including nutrition, exercise, smoking, alcohol use, sexuality and vaccination); 12 on maternity in general (e.g. partners, family, social support, family planning, legislation and adolescent mothers); 20 on pregnancy, from its physiology to health problems, covering perinatal death as well as fears and concerns; 14 on labor and the birth, covering choice of where to give birth, its physiology, pain management, and health problems, again including fears and concerns; 4 on the postpartum period; 4 on nutrition of the newborn (breastfeeding and bottle feeding), and 16 on care of the newborn and infants under 12 months of age. (Appendix 1).

### Prioritisation of topics using the Delphi method

**Fig. 1:**
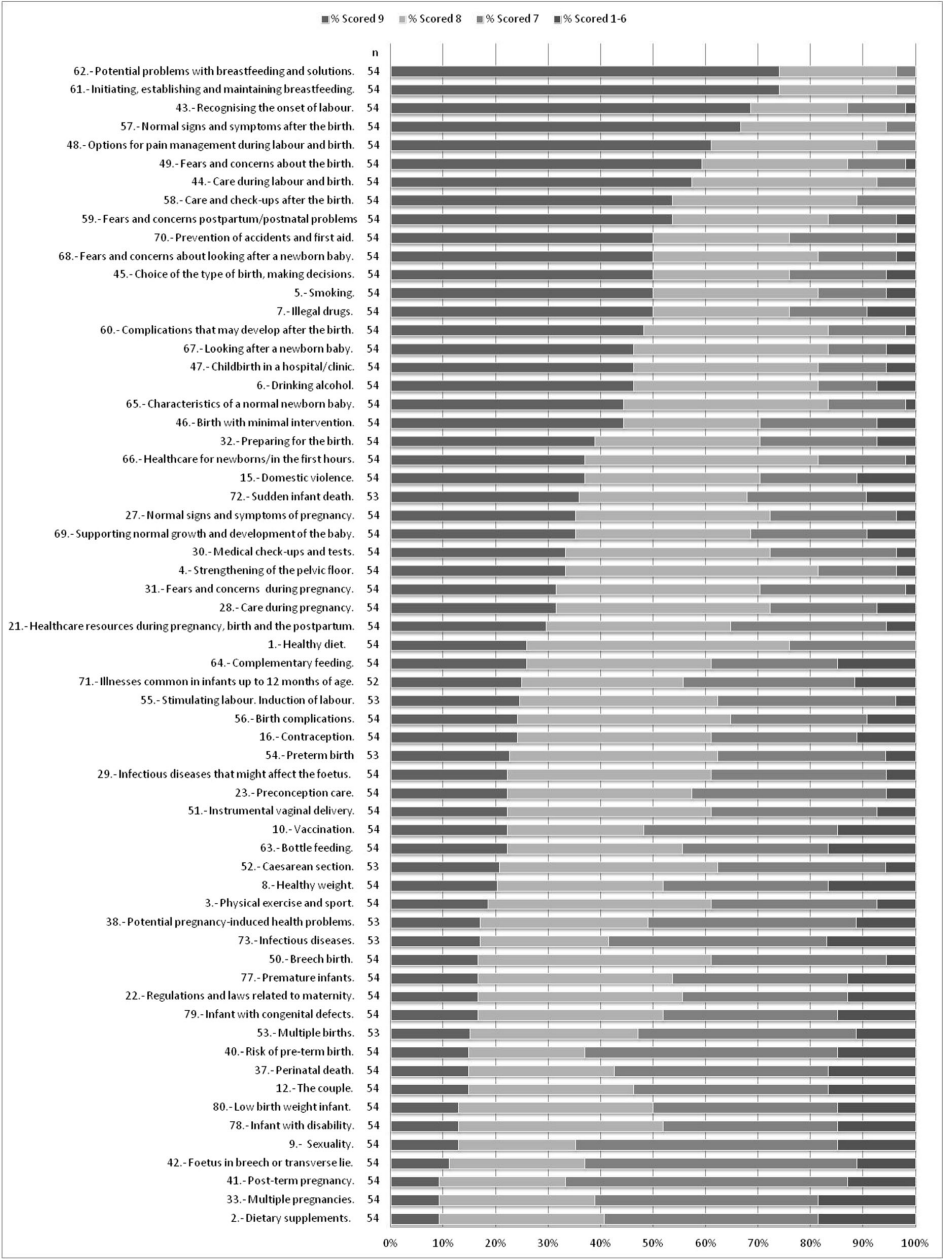
List of the 63 items considered “important” by at least 80% of participants. The colors indicate the percentage of participants who awarded each score or range of scores to each item

For the first round of the Delphi study, we sent the questionnaire to a total of 356 people, 117 health professionals and 239 non-healthcare professionals, and received 79 completed questionnaires; 22.2% of all the questionnaires sent. In the second round, we sent the questionnaire to the individuals who had answered the first round and received 54 questionnaires. As reflected in Table 1, 84.8% of the questionnaires sent were not answered. Most participants who responded were healthcare professionals (42/54). A total of 63 topics were considered important (scores of 7 to 9) by more than 80% of the participants (Fig. 1). None of the topics were considered unimportant, and Table 2 indicates those that ranked the lowest. In the light of the high number of topics considered important, we decided to select only those assigned scores of 8 or 9 by at least 80% of participants (18 topics) or scores of 7 to 9 by at least 95% of participants (a further 6 topics). In this way, as shown in Table 3, we finally selected 24 topics: 13 related to maternity, pregnancy and childbirth and 11 to the postpartum period, breastfeeding, childrearing, and the newborn and young infant.

**Table 1:** Experts invited to participate and those who participated in the Delphi study

Experts		No. of questionnaires sent, 1st round	No. of questionnaires received, 1st round (= sent, 2nd round)	No. of questionnaires received, 2nd round	Final response rate %
Health professionals	Midwives	70	38	23	32.9
	Obstetrician/gynecologists	13	10	9	69.2
	Pediatricians	16	6	6	37.5
	Pediatric nurses	18	4	4	22.2
Non-health professionals	Women’s association representatives	42	15	10	23.8
	State school staff		0	0	0.0
	Private sector school staff	107	5	2	1.9
	Nursery staff	46	1	0	0.0
TOTAL		356	79	54	15.2

**Table 2:** Topics that obtained the lowest scores in the Delphi study

Topic	Number of participants who Scored 0–6	% of Participants
74.- Infants with behavioral problems.	11	20.3
76.- Twins.	11	20.3
75.- Food allergies.	12	22.2
20.- Adolescent mothers.	13	24.0
25.- Difficulties conceiving.	13	24.0
36.- Fetal defects and abnormalities.	13	24.0
35.- Miscarriage.	14	25.9
17.- Sexually transmitted infections.	14	26.4
13.- The family.	15	27.7
14.- Social and community support.	15	27.7
18.- Motherhood as a single parent.	15	27.7
19.- Families with same-sex parents.	15	27.7
11.- Body changes related to reproduction.	16	29.6
39.- Intrauterine growth restriction.	16	29.6
26.- Sterility/infertility treatment.	17	31.4
34.- Ectopic pregnancy.	21	38.8
24.- Pregnancy testing: when and how.	23	42.5

**Table 3:** List of the 24 most highly ranked topics in Delphi analysis. *n* = 54 participants^a^

Topic	Participants who Scored ≥7	%	Participants who Scored ≥8	%
1.- Healthy diet.	54	100.0	41	75.9
4.- Strengthening the pelvic floor.	52	96.30	44	81.4
5.- Smoking.	51	94.44	44	81.4
6.- Drinking alcohol.	50	92.59	44	81.4
27.- Normal signs and symptoms of pregnancy.	52	96.30	39	72.2
30.- Medical check-ups and tests.	52	96.30	39	72.2
31.- Fears and concerns during pregnancy.	53	98.15	38	70.3
43.- Recognizing the onset of labor.	53	98.15	47	87.0
44.- Care during labor and birth.	54	100.0	50	92.5
47.- Childbirth in a hospital/clinic.	51	94.44	44	81.4
48.- Options for pain management during labor and birth.	54	100.0	50	92.5
49.- Fears and concerns about the birth.	53	98.15	47	87.0
55.- Stimulating labor. Induction of labor. ^a^(Total *n* = 53)	51	96.23	33	62.3
57.- Normal signs and symptoms after the birth.	54	100.0	51	94.4
58.- Care and check-ups after the birth.	54	100.0	48	88.8
59.- Fears and concerns postpartum/postnatal problems.	52	96.30	45	83.3
60.- Complications that may develop after the birth.	53	98.15	45	83.3
61.- Initiating, establishing and maintaining breastfeeding.	54	100.0	52	96.3
62.- Potential problems with breastfeeding and solutions.	54	100.0	52	96.3
65.- Characteristics of a normal newborn baby.	53	98.15	45	83.3
66.- Healthcare for newborns in the first hours.	53	98.15	44	81.4
67.- Looking after a newborn baby.	51	94.44	45	83.3
68.- Fears and concerns about looking after a newborn baby.	52	96.30	44	81.4
70.- Prevention of accidents and first aid.	52	96.30	41	75.9

**Fig. 2:**
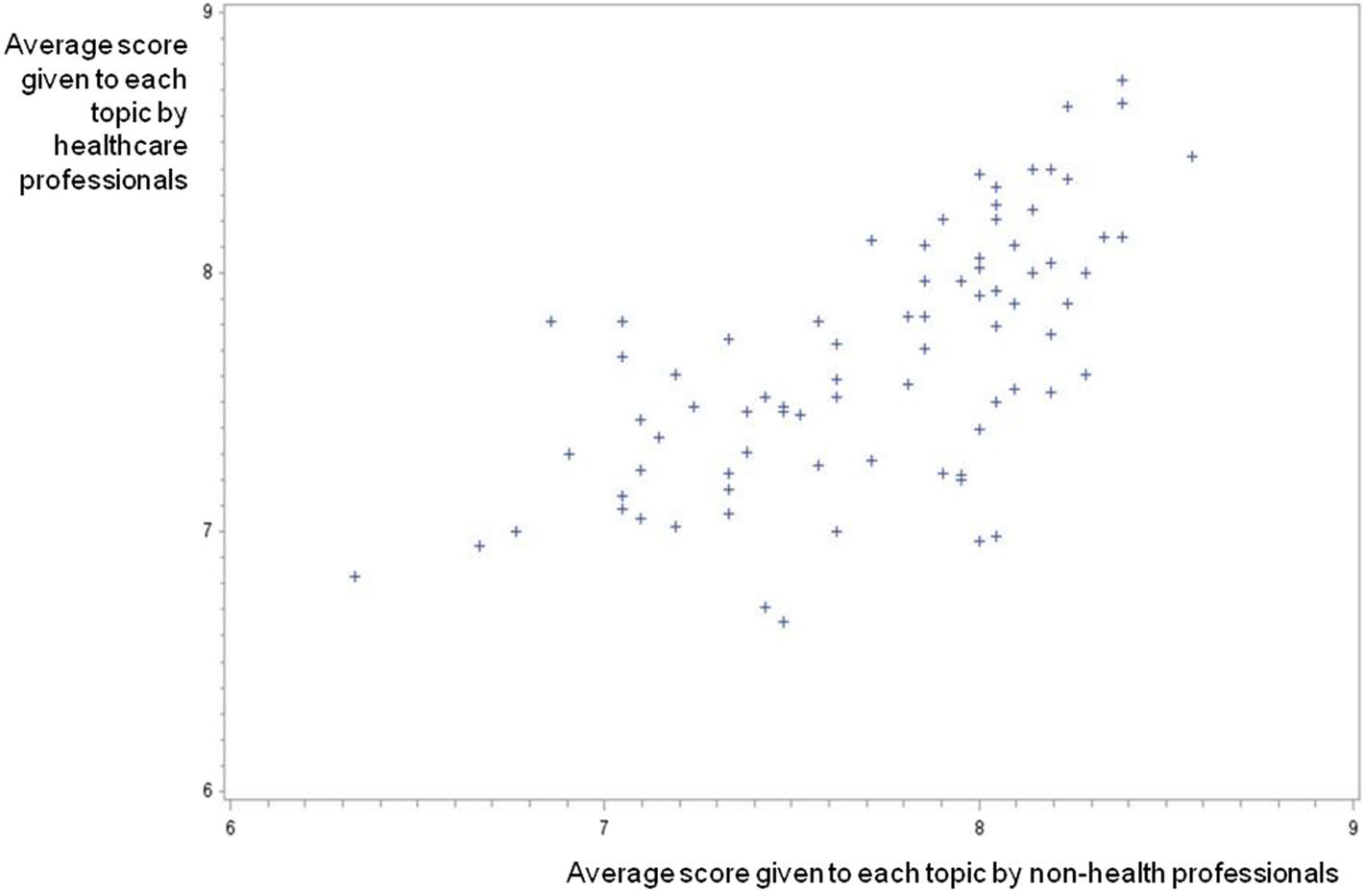
Correlation between rankings of health and non-health experts on the Delphi questionnaire

Healthcare and non-healthcare participants were assigned different encoding to enable us to compare the scores given by both groups and we did not find significant differences between the topics selected by the healthcare and non-healthcare groups, while there was a strong association between the rankings given by each group (Spearman’s correlation coefficient 0.68064, *p* < .0001, Fig. 2).

Certain specific topics stood out as being mentioned frequently in the first survey, including information on community-based resources for support; the need to include the father in the pregnancy, childbirth and postpartum processes; maternal support in the postpartum period; special situations during childrearing (adopted children, cultural practices such as circumcision and female genital mutilation); and the need to emphasize alternative approaches for coping with childbirth.

### Seeking consensus among experts: the nominal group technique

We created an expert group, including 12 relevant professionals as well as 2 members of the research team, to identify the five most important topics for maternal education. Only one person initially recruited to this group was unable to participate, a pediatrician, and she was replaced by a male colleague. The final group was made up of nine women and three men, eight of whom were healthcare professionals and four of whom non-healthcare professionals.

In the discussions, as well as the 24 topics that scored the highest in the Delphi study, we invited the experts to bring up any of the topics that had been excluded but that they might personally consider very important. In this way, the topics of “Social and community support” for mothers (topic 14*)* and “Choice of the type of birth” (topic 45) were re-included. As well as these, it was noted that the initial list did not mention “immigration in our area” as a potential determinant in all the topics. The following is a summary of each of the sessions, with sample quotes from participants’ comments.

#### Session 1: topics related to maternity, pregnancy and childbirth

Participants discussed the difficulty of further dividing or specifying the topics, given that they felt some to be closely related:

*“For me, topics 48, 49 and 46 should be considered together, and as a midwife, I’m unable to talk about them separately: it’s all to do with supporting normal physiological childbirth” (G)*

- Nutrition was mentioned but always together with lifestyle, which was mentioned eight times:

*“It doesn’t seem possible to me to talk about nutrition without mentioning alcohol, or exercise without mentioning smoking” (G)*

- Similarly, the normal signs of pregnancy were grouped with resources for change management, and the building of skills at this stage, mentioned by seven participants.
- Signs of the onset of labor were mentioned by two participants.
- Participants considered a topic covering the development of a birth plan and related decision-making to be a very important topic, which involved knowing the options available and taking responsibility, this being mentioned by eight people:

*“… choice of the type of birth, development of a birth plan (a template being given to women between weeks 28 and 32 of pregnancy); people’s rights and duties, informed consent. It is important that best practice indicators are clear” (E).*

- The topic of pain management during labor and birth, existing options and empowerment at this stage was mentioned by eight people:
- Resources available in the community was mentioned twice.
- Care for immigrants and fear of giving birth were mentioned by one participant each.

#### Session 2: topics related to the postpartum period, breastfeeding and newborn care

- On six occasions, the normal symptoms of the postpartum were mentioned, frequently in conjunction with care and rehabilitation of the pelvic floor, which was mentioned by five participants. The ‘baby blues’ were also mentioned:

*“We talk more about the physical aspects, but we women don’t talk about what we feel after giving birth and you feel like you’re going crazy… it turns out that we are all in the same boat and that it doesn’t usually last long, but it seems like it’s more stigmatized if you look for help …”* (E)

- All the participants considered the initiation of breastfeeding to be very important, in many cases without being able to separate it from the establishment of breastfeeding. They talked particularly about the fact that the information should be given at the right time, observing the lack of effectiveness of this information if the woman is not actually breastfeeding.
- Solving of the most common problems during breastfeeding was mentioned by seven participants:

*“They feel accompanied by midwives during pregnancy but afterwards they feel abandoned, because pediatricians also care for the baby, and of course… that’s all very well… but all their needs… We joke that breasts are left in no-man’s land”.*

- While three participants mentioned the topic of describing what a normal newborn baby is like, nine participants referred to the topic of “looking after a newborn baby”, stating that the information on care involved already having information about what could be considered normal, thereby linking the two topics.
- Two participants mentioned supplementary feeding of newborn babies, and the following topics were mentioned once: perinatal grief and mourning, nonviolent communication, the role of the father, searching for support, problems with the extended family, bottle feeding, community resources and care for mild infant illnesses.

#### Session 3: Selection of the 5 highest priority topics for maternal education

During the third session, after the participants’ proposals and discussions, the voting process started, with participants ordering the 5 topics they considered most important (ranking each, giving 5 to the most important and 1 to the least important). The participants considered “initiating and maintaining breastfeeding” and “problems with breastfeeding and solutions” to be inseparable, and these were therefore considered a single topic in the voting. Similarly, they grouped “normal signs and symptoms after the birth”, “care and check-ups after the birth” and “strengthening of the pelvic floor” into one topic; and “characteristics of a normal newborn” and “looking after a newborn” into another topic. Finally, we were left with eight topics that were considered essential and ranked as shown in Table 4.

**Table 4:** List of the topics prioritized in the from nominal group technique and score obtained

Topic	Score	% of total score awarded
Initiating, establishing and maintaining breastfeeding. Common problems and potential solutions.	50	27.7
Choice of the type of birth, making decisions: The birth plan	44	24.4
Normal signs and symptoms after the birth/care and check-ups/pelvic floor rehabilitation	31	17.2
Diet (always mentioned together with a healthy lifestyle)	24	13.3
Options for pain management during labor and birth	14	7.7
Characteristics of a normal newborn/looking after a newborn baby	12	6.6
Normal signs and symptoms of pregnancy	3	0.0
Social and community support	2	0.0

## Discussion

The goal of maternal education, which originally was to reduce pain during labor and childbirth, has nowadays become a way of fostering a smooth transition towards motherhood. This goal, which is very broad and sometimes too vague, needs to be interpreted more specifically in terms of situations and contents. There have been some trials of more intensive interventions which focused on certain topics, assessing the benefits obtained, with mixed results [14]. This study has responded to our research question, identifying the topics are a priority for women here and which merit more intensive and extensive intervention [17], with the aim of developing an intervention that would be specifically focused on priority needs and testing its efficacy, as is recommended for complex intervention models [21, 22].

The results obtained in this study indicate that the topics on which most emphasis should be placed in maternal education and for which results obtained should be evaluated are: care during the initiation and establishment of breastfeeding; information for shared decision-making with regards to childbirth; identification of problems in the postpartum period and coping tools; advice on and reinforcement of a healthy lifestyle throughout the entire process; and information and training on various options for pain management during labor and birth, beyond the use of epidural anesthesia.

The results coincide with those mentioned by Stevens et al. [43], McKinnon et al. [44] or Downer [45] in relation to coping with childbirth, the possibilities of mobility, use of medical procedures or place of delivery. In this study, women have expressed a general need for information and support in decision-making regarding pregnancy or breastfeeding, which will allow them to play a more active role in the process. Despite significant socioeconomic differences, some information needs are similar across countries and coincide with those mentioned in this paper, such as the interest in pregnancy care, to deal adequately with childbirth, and the care of the newborn. However, there are also notable differences; for example, the results obtained by Chicalipo et al. [15] with African populations the priorities are more oriented towards the prevention of illness. Compared with Chicalipo’s study, the priorities in western countries are aimed at improving the quality of life and self care. No doubt these differences will be related to the difference in resources, but also to the social and cultural context.

Unlike that study, this work gives priority to information and support for self-care of the puerperal woman and the beginning of breastfeeding. It is possible that this emphasis in our data is related to cultural habits in the postpartum, so that women who go through this stage in extended families, with the support of other women, do not perceive this need in the same way as women living in nuclear families, who go through this new experience alone [46, 47].

These results are to a great extent consistent with the needs identified from Internet searches. During pregnancy, the terms most commonly searched for include nutrition, having a healthy lifestyle and detecting potential complications [10, 16, 32], both by mother and fathers [48]. The concerns of women vary during pregnancy [9], and when childbirth is closer, the searches become focused on the start and progress of labor, as well as potential associated complications [49]. In many cases, the evidence indicates an intention to take an active role, with searches on topics such as options for pain management, apart from epidural anesthesia [50]. We have also found agreement on priority topics after childbirth between the results of our study and Internet searches, especially with regards to breastfeeding [13, 17, 51] and looking after a newborn [48]. Concerning the post-partum period, there is also mention of the need for maternal care in general and information on maternal self-care and on episiotomy care in particular; of getting back into shape and of sexuality, and probably most importantly, of the need to share experiences between peers and the availability of psychological support at this stage [52].

Therefore, a maternal education program that seeks to be effective for the population of our environment, should work on these specific topics, investing time and resources, analyzing and assessing the specific needs of each woman, proposing actions focused on modifying the situation and evaluating the results obtained. Only this way we will be able to achieve positive and measurable results in women’s health and satisfaction [22]. The next question would be how to assess and respond to these needs. Group-based interventions on specific topics have been shown to be effective in some areas such as reducing the intake of toxic substances and increasing the uptake of breastfeeding [14]. On the other hand, not all women have the time or availability necessary to attend such activities [53]. New technologies have also been shown to be useful, such as in supporting longer breastfeeding [54] and building self-confidence in taking decisions regarding labor or childrearing [55]. Indeed, IT resources offer potentially effective support tools that make it possible to assess the needs expressed and offer an accessible and immediate response, as well as allowing parents to share experiences and establish support groups [56]. Digital tools can be detailed, entertaining, customized, practical, professional, reassuring and unbiased [33].

The results of this work support the view that there have been changes in the attitudes of professionals and women towards the experience of pregnancy, childbirth and the postpartum period, as observed in other studies [44, 57]. Women demonstrate a clear need to lead their health and professionals are interested in empowering women’s self-care, giving them a leading role of a physiological process, such as motherhood. The woman is seen as an active subject, interested in expressing her wishes and needs and concerned about behaving in the most appropriate way to care for her health and that of her child. Though some women continue to feel that a medicalized birth is healthier [58], they are no longer passive receptors of information; rather, they actively seek out the information that they find most useful [59] and make choices concerning the birth based on their pre-existing beliefs [60]. Their attitude is proactive, and they seem to be more interested in advice to help them improve their behavior than in passively acquired information. All the topics prioritized in our study involve decision-making and the behavior of the women themselves.

Some of the topics considered a high priority in this study, for example, breastfeeding, are often addressed on the most widely consulted websites, both in terms of the number of websites concerning this specific topic, and the importance given to this topic on websites focusing on pregnancy and childcare [36]. Furthermore, an emphasis on nutrition and a healthy lifestyle during pregnancy, and information on childcare can be found on many websites on child health. Nevertheless, there are fewer tools which women can use to reflect on their wishes regarding the labor process, or on their experiences of the postpartum period, their personal and/or family situation, their relationship with their child, and so on. Some initiatives in these areas propose the use of technological tools for accompanying women and providing them with advice in the postpartum period via videoconferences or contact between peers, approaches that merit thorough research to assess their impact on different parts of the population and their efficacy in improving the well-being of couples and their children [55]. The work presented here will allow for the design of tools that evaluate and respond to the highlighted needs; it will hasten the application of such resources among the population and will enable researchers to analyze their effect using randomized controlled trials, following the methodology proposed by recent models of research in implementation. [61]

### Strengths and limitations

This study adds to our knowledge concerning topics that should be addressed specifically in maternal education. The method used, with an initial selection and a subsequent in-depth analysis with participants who had experience in all the relevant areas, has been rigorous and reinforces the results. The inclusion of a group of participants from outside the healthcare professions provides a point of view that may more closely reflect the needs of women in their daily life.

Nonetheless, this study also has certain limitations. The participation rate of people invited to carry out the Delphi study was low. It is likely that individuals who participated were more motivated and this may be a source of bias in the results. On the other hand, if we exclusively consider healthcare professionals and representatives of women’s associations, the response rate was 34.7%, which is comparable to that found in other similar studies, such as those of Slomian (35.9%) [13]. The almost total lack of participation of representatives of school associations may be attributable to the fact when children start to attend pre-school, at 2 to 3 years of age, pregnancy, birth and the postpartum period seem distant, with little relevance once they have passed. Furthermore, the individuals who participated in the nominal group workshop were selected based on the opinions of their colleagues, considering their length of experience and their previous involvement in clinical innovation, research and/or teaching, and we believe that they brought a significant level of expertise to the discussions. We did not, however, apply strictly objective criteria and we recognize that the most active people are not necessarily the most knowledgeable.

Moreover, it is difficult to generalize the results, given that all the participants came from the same geographical area (Basque Country). Nevertheless, it is justifiable to collect such site-specific data when the aim is to assess needs or priorities in a particular health context [10].

## Conclusion

Maternal education should focus on specific aspects of health, namely, ones that are particularly important for women at each stage, if we want to achieve effective intervention. Personal support and accompaniment by health professionals and peers is essential during pregnancy, labor, birth and the postpartum period, but the use of digital tools in maternal education can provide new resources, especially given the immediacy of response they can provide and the fact that they can help overcome people’s limitations in terms of time and availability. This would facilitate the empowerment of women and self-management of their health. Internet tools are commonly used by women from all social strata [54, 62] and in some cases they represent the main source of information on pregnancy and the postpartum period, such as in the case of highly qualified and/or immigrant women, for whom going to maternal education sessions is not always feasible [8, 53].

Some of the priority topics indicated in this study, such as breastfeeding, baby care and nutrition during pregnancy have been addressed through primary care or pediatric services, and a wide range of digital materials is already available. Nevertheless, it is necessary to develop, test and evaluate instruments to assess and respond to the needs of women in other areas prioritized in this study, such as regarding their preferences in labor and how to cope with the postpartum period. Therefore, the midwife must take on the role of advising on the quality of information available on the Internet, include this resource in their daily work and generate digital tools to adapt to the needs demanded by women.

## Data Availability

The datasets used and/or analysed during the current study available from the corresponding author on reasonable request.

## References

[1] Fuentes-Pelaez N, Molina MC, Amoros P, Jane-Checa M, Martinez-Bueno C (2013). The design of a maternal education program based on analysis of needs and collaborative work. Rev Cercet Interv So.

[2] Stevens G, Miller YD (2012). Overdue choices: how information and role in decision-making influence women's preferences for induction for prolonged pregnancy. Birth-Iss Perinat C.

[3] Wittmeier KD, Hobbs-Murison K, Holland C, Crawford E, Loewen H, Morris M, Lum Min S, Abou-Setta A, Keijzer R (2018). Identifying information needs for Hirschsprung disease through caregiver involvement via social media: a prioritization study and literature review. J Med Internet Res.

[4] Akca A, Corbacioglu EA, Ozyurek ES, Aydin A, Korkmaz N, Gorgen H (2017). The influence of the systematic birth preparation program on childbirth satisfaction. Arch Gynecol Obstet.

[5] Truccolo Ivana, Mazzocut Mauro, Cipolat Mis Chiara, Bidoli Ettore, Zotti Paola, Flora Silvia, Mei Luigina, Apostolico Mauro, Drace Christina, Ravaioli Valentina, Conficconi Alice, Cocchi Simone, Cervi Elena, Gangeri Laura, De Paoli Paolo (2018). Patients and caregivers’ unmet information needs in the field of patient education: results from an Italian multicenter exploratory survey. Supportive Care in Cancer.

[6] Frenk J, Chen L, Bhutta ZA, Cohen J, Crisp N, Evans T (2010). Health professionals for a new century: transforming education to strengthen health systems in an interdependent world. Lancet..

[7] Deave T, Johnson D, Ingram J (2008). Transition to parenthood: the needs of parents in pregnancy and early parenthood. BMC Pregnancy Childbirth..

[8] Svensson J, Barclay L, Cooke M (2006). The concerns and interests of expectant and new parents: assessing learning needs. J Perinat Educ.

[9] Paz-Pascual C, Artieta-Pinedo I, Grandes G, Espinosa-Cifuentes M, Gaminde-Inda I, Payo-Gordon J (2016). Necesidades percibidas por las mujeres respecto a su maternidad. Estudio cualitativo para el rediseño de la educación maternal. Aten Prim.

[10] Almalik MMA, Mosleh SM (2017). Pregnant women: what do they need to know during pregnancy? A descriptive study. Women Birth.

[11] Gazmararian JA, Dalmida SG, Merino Y, Blake S, Thompson W, Gaydos L (2014). What new mothers need to know: perspectives from women and providers in Georgia. Matern Child Health J.

[12] Walker LO, Murphey CL, Nichols F (2015). The broken thread of health promotion and disease prevention for women during the postpartum period. J Perinat Educ.

[13] Slomian J, Emonts P, Erpicum M, Vigneron L, Reginster JY, Bruyère O (2017). What should a website dedicated to the postnatal period contain? A Delphi survey among parents and professionals. Midwifery.

[14] McMillan AS, Barlow J, Redshaw M. Birth and Beyond: A Review of Evidence about Antenatal Education. Department of Health London website https://shu.rl.talis.com/items/A003BF1B-97F9-CB2F-525C-52C2F1FD5ABA.html Published 2009. Accessed 2 July 2019.

[15] Chikalipo MC, Chirwa EM, Muula AS (2018). Exploring antenatal education content for couples in Blantyre, Malawi. BMC Pregnancy Childbirth.

[16] Sayakhot P, Carolan-Olah M (2016). Internet use by pregnant women seeking pregnancy-related information: a systematic review. BMC Pregnancy Childbirth.

[17] Almalik MM (2017). Understanding maternal postpartum needs: a descriptive survey of current maternal health services. J Clin Nurs.

[18] Buultjens M, Murphy G, Robinson P, Milgrom J, Monfries M (2017). Women's experiences of, and attitudes to, maternity education across the perinatal period in Victoria, Australia: a mixed-methods approach. Women Birth..

[19] Heim MA, Miquelutti MA, Makuch MY. Perspective of pregnant women regarding antenatal preparation: A qualitative study. Women Birth. 2018. 10.1016/j.wombi.2018.11.016 [Epub ahead of print]. 10.1016/j.wombi.2018.11.016 30528818

[20] Deliktas D, Kukulu K, Haugan G. Aune I. “I want a birth without interventions”: Women's childbirth experiences from Turkey. Women Birth. 2018. 10.1016/j.wombi.2018.12.011 [Epub ahead of print]. 10.1016/j.wombi.2018.12.011 30600167

[21] Aventin Á, Lohan M, O'Halloran P, Henderson M (2015). Design and development of a film-based intervention about teenage men and unintended pregnancy: applying the Medical Research Council framework in practice. Eval Program Plann.

[22] Craig P, Dieppe P, Macintyre S, Michie S, Nazareth I, Petticrew M (2008). Developing and evaluating complex interventions: the new Medical Research Council guidance. BMJ.

[23] Dane AC, Peterson M, Miller YD (2018). Talking points: Women's information needs for informed decision-making about noninvasive prenatal testing for Down syndrome. J Genet Couns.

[24] Abuidhail J, Mrayan L, Jaradat D (2019). Evaluating effects of prenatal web-based breastfeeding education for pregnant mothers in their third trimester of pregnancy: prospective randomized control trial. Midwifery..

[25] Campbell V, Nolan M (2019). ‘It definitely made a difference’: a grounded theory study of yoga for pregnancy and women's self-efficacy for labour. Midwifery..

[26] Mosleh SM, Eshah NF, Almalik M (2017). Perceived learning needs according to patients who have undergone major coronary interventions and their nurses. J Clin Nurs.

[27] Cipolat Mis C, Truccolo I, Ravaioli V, Cocchi S, Gangeri L, Mosconi P, Drace C, Pomicino L, Paradiso A, De Paoli P (2015). Italian Cancer patient education making patient centered care a reality: a survey of patient educational programs in Italian Cancer research and care institutes. BMC Health Serv Res.

[28] Van Teijlingen E, Pitchforth E, Bishop C, Russell E. Delphi method and nominal group technique in family planning and reproductive health research. BMJ website. https://srh.bmj.com/content/32/4/249.long. Published 2006. Accessed 2 July 2019. 10.1783/147118906778586598 17032518

[29] Siegfried AL, Carbone EG, Meit MB, Kennedy MJ, Yusuf H, Kahn EB (2017). Identifying and prioritizing information needs and research priorities of public health emergency preparedness and response practitioners. Disaster Med Public Health Prep.

[30] EUSTAT. Euskal Estatistika Erakundea / Instituto Vasco de Estadística website. http://www.eustat.eus/elementos/ele0000000/ti_Sector_hospitalario_Recursos_actividad_asistencial_y_economica_por_territorio_historico_titularidad_y_tipo_de_hospital_2010/tbl0000069_c.html Published 2018. Accessed 2 July 2019.

[31] Neter E, Brainin E (2012). eHealth Literacy: Extending the Digital Divide to the Realm of Health Information. J Med Internet Res.

[32] Lagan BM, Sinclair M, Kernohan WG (2010). Internet use in pregnancy informs women's decision making: a web-based survey. Birth-Iss Perinat C.

[33] Lupton D (2016). The use and value of digital media for information about pregnancy and early motherhood: a focus group study. BMC Pregnancy Childbirth.

[34] Wiener RC, Wiener-Pla R (2014). Literacy, pregnancy and potential oral health changes: the internet and readability levels. Maternal Child Health J.

[35] Minervation, The Lida instrument: minervation validation instrument for healthcare websites. http://www.minervation.com/wp-content/uploads/2011/04/Minervation-LIDA-instrument-v1-2.pdf. Accessed 12 May 2014.

[36] Artieta-Pinedo I, Paz-Pascual C, Grandes G, Villanueva G, Ema Q Group (2018). An evaluation of Spanish and English on-line information sources regarding pregnancy, birth and the postnatal period. Midwifery..

[37] Boulkedid R, Abdoul H, Loustau M, Sibony O, Alberti C (2011). Using and reporting the Delphi method for selecting healthcare quality indicators: a systematic review. PLoS One.

[38] Okoli C, Pawlowski SD (2004). The Delphi method as a research tool: an example, design considerations and applications. Inform Manag.

[39] Brown BB (1968). Delphi process: a methodology used for the elicitation of opinions of experts: an earlier paper published by RAND. Document No: P-3925.

[40] Artieta-Pinedo I, Paz-Pascual C, Grandes G (2017). Framework for the establishment of a feasible, tailored and effective perinatal education programme. BMC Pregnancy Childbirth.

[41] Emakunde. Guía de Asociaciones de Mujeres en la Comunidad Autónoma de Euskadi. Emakunde/Instituto Vasco de la Mujer. http://www.emakunde.euskadi.eus/contenidos/informacion/publicaciones_guias2/es_emakunde/adjuntos/guia_asoc_mujer_euskadi.pdf. Published 2014. Accessed 2 July 2019.

[42] Stolper E, van Leeuwen Y, Van Royen P, van de Wiel M, van Bokhoven M, Houben P (2010). Establishing a European research agenda on ‘gut feelings’ in general practice. A qualitative study using the nominal group technique. Eur J Gen Pract.

[43] Stevens G, Miller YD, Watson B, Thompson R (2016). Choosing a Model of Maternity Care: Decision Support Needs of Australian Women. Birth..

[44] McKinnon LC, Prosser SJ, Miller YD (2014). What women want: qualitative analysis of consumer evaluations of maternity care in Queensland, Australia. BMC Pregnancy Childbirth.

[45] Downer T (2018). Exploring antenatal education: an interpretive description.

[46] Chen CH (2015). Revision and validation of a scale to assess pregnancy stress. J Nurs Res.

[47] Srkalović Imširagić A (2017). Prediction of posttraumatic stress disorder symptomatology after childbirth - A Croatian longitudinal study. Women Birth.

[48] Oscarsson MG, Holmström I, Medin E, Lendahls L (2018). Using the internet as source of information during pregnancy-a descriptive cross-sectional study among fathers-to-be in Sweden. Midwifery..

[49] Javanmardi M, Noroozi M, Mostafavi F, Ashrafi-rizi H (2018). Internet usage among pregnant women for seeking health information: a review article. Iran J Nurs Midwifery Res.

[50] Sutton CD, Carvalho B (2017). What's trending now? An analysis of trends in internet searches for labor epidurals. Int J Obstet Anesth.

[51] Slomian J, Bruyère O, Reginster JY, Emonts P (2017). The internet as a source of information used by women after childbirth to meet their need for information: a web-based survey. Midwifery..

[52] Slomian J, Emonts P, Vigneron L, Acconcia A, Glowacz F, Reginster JY, Bruyère O (2017). Identifying maternal needs following childbirth: a qualitative study among mothers, fathers and professionals. BMC Pregnancy Childbirth..

[53] Romano AM (2007). A changing landscape: implications of pregnant women's internet use for childbirth educators. J Perinat Educ.

[54] Newby R, Brodribb W, Ware RS, Davies PS (2015). Internet use by first-time mothers for infant feeding support. J Hum Lact.

[55] Slomian Justine, Emonts Patrick, Vigneron Lara, Acconcia Alessandro, Reginster Jean-Yves, Oumourgh Mina, Bruyère Olivier (2017). Meeting the Needs of Mothers During the Postpartum Period: Using Co-Creation Workshops to Find Technological Solutions. JMIR Research Protocols.

[56] van Dijk MR, Oostingh EC, Koster MP, Willemsen SP, Laven JS, Steegers-Theunissen RP (2017). The use of the mHealth program smarter pregnancy in preconception care: rationale, study design and data collection of a randomized controlled trial. BMC Pregnancy Childbirth..

[57] Benyamini Y, Molcho ML, Dan U, Gozlan M, Preis H (2017). Women’s attitudes towards the medicalization of childbirth and their associations with planned and actual modes of birth. Women Birth..

[58] Hunter A, Devane D, Houghton C, Grealish A, Tully A, Smith V (2017). Woman-centred care during pregnancy and birth in Ireland: thematic analysis of women’s and clinicians’ experiences. BMC Pregnancy Childbirth..

[59] Pullon S, Ballantyne A, Macdonald L, Barthow C, Wickens K, Crane J. Daily decision-making about food during pregnancy: a New Zealand study. Health Promotion International, 2018; 1-10. dax098, 10.1093/heapro/dax098. 10.1093/heapro/dax098 29342272

[60] Preis H, Gozlan M, Dan U, Benyamini Y (2018). A quantitative investigation into women's basic beliefs about birth and planned birth choices. Midwifery..

[61] Wu JP, Damschroder LJ, Fetters MD, Zikmund-Fisher BJ, Crabtree BF, Hudson SV, Ruffin MT, Fucinari J, Kang M, Taichman LS, Creswell JW (2018). A Web-Based Decision Tool to Improve Contraceptive Counseling for Women with Chronic Medical Conditions: Protocol For a Mixed Methods Implementation Study. JMIR Res Protoc.

[62] Zimmerman MS (2018). Assessing the reproductive health-related information-seeking behavior of low-income women: describing a two-step information-seeking process. Health Commun.

